# Editorial: Immunosuppression mechanisms and immunotherapy strategies in glioblastoma

**DOI:** 10.3389/fncel.2024.1411330

**Published:** 2024-04-25

**Authors:** Sihan Xiong, Bing Qin, Chuang Liu, Yuanbo Pan

**Affiliations:** ^1^Department of Pathology, Brigham and Women's Hospital, Harvard Medical School, Boston, MA, United States; ^2^Department of Neurosurgery, Second Affiliated Hospital, School of Medicine, Zhejiang University, Hangzhou, China; ^3^Department of Neurosurgery, Brigham and Women's Hospital, Harvard Medical School, Boston, MA, United States

**Keywords:** glioblastoma (GBM), immunotherapy, immunosuppression, tumor microenvironment (TME), blood-brain barrier (BBB)

Gliomas are brain tumors that arise from neuroglial progenitor cells in the brain, which have an annual incidence rate of around six per 100,000 people in the US (Ostrom et al., 2013). Glioblastoma (GBM) is the most aggressive type of glioma and comprises about half of all glioma cases (Ostrom et al., 2013). In patients diagnosed with GBM, the median survival of only 15 months is expected when they receive temozolomide (TMZ), a chemotherapy medicine, with postoperative radiotherapy (RT) (Stupp et al., 2005; Koshy et al., 2012; Ostrom et al., 2013). GBM resides in a crucial organ that can complicate the treatment, and the characteristic immunosuppressive tumor microenvironment (TME) shielded by the blood-brain barrier (BBB) can hinder immunotherapy and drug delivery to the brain (Bellail et al., 2004; Quail and Joyce, 2017; Lim et al., 2018). For instance, combining chemotherapeutic temozolomide (TMZ) with radiation therapy enhances patient survival, but may lead to a TME re-modeling process that promotes a resistant, pro-invasive tumor phenotype (Stupp et al., 2005; Franceschi et al., 2009). GBM cells can also respond to radiation by increasing hyaluronic acid (HA) production or activating transcription factors that resist further radiation and increase subsequent invasiveness (Akiyama et al., 2001; Rath et al., 2015; Yoo et al., 2018).

To tackle these problems, numerous therapy strategies and drugs have been extensively tested in GBM treatment (Lim et al., 2018; Wolf et al., 2019). For instance, immune-checkpoint blockades (ICBs) are a well-researched immunotherapy strategy, and inhibitors such as anti-cytotoxic T lymphocyte-associated protein 4 (CTLA-4)/anti-programmed cell death protein 1 (PD-1) and anti-programmed cell death protein ligand 1 (PD-L1) in other types of cancers have been considered for treating GBM. Bevacizumab antibody works by blocking vascular endothelial growth factor (VEGF) and is approved for recurrent glioblastoma in various countries, including the US, while nivolumab (anti-PD-1) is a low-toxicity ICB that has been studied alone and in combination with ipilimumab (anti-CTLA-4) (Weller et al., 2017a; Lim et al., 2018; Reardon et al., 2020). Furthermore, oncolytic virus as an anticancer therapy has seen progress in the use of poliovirus, adenoviruses, and parvovirus alike (Lim et al., 2018). One example is AdvHSV-tk (adenovirus/herpex simplex-thymidine kinase), an adenoviral vector that delivers herpes simplex virus type 1 (HSV-1) into tumor cells, with phase I and II studies demonstrating its ability to elicit tumor apoptosis or necrosis when ganciclovir is co-administered (Immonen et al., 2004; Wheeler et al., 2016; van Solinge et al., 2022). Morever, the most advanced vaccination therapy is Rindopepimut^®^ (also known as CDX-110); it mimics and targets an antigen called EGFRvIII (epidermal growth factor receptor variant III) that expresses in 25%−30% of primary GBM (Weller et al., 2014, 2017b; Lim et al., 2018). Rindopepimut is well tolerated, can induce an immune response in favorably selected patients, and can potentially improve the survival of those with significant residual disease, if suitable combinatorial approaches are applied (Schuster et al., 2015; Weller et al., 2017b). Additionally, chimeric antigen receptors (CARs) are synthetic constructs expressed by engineered T cells and represent another well-researched immunotherapy. CAR T cells can recognize antigens independently of the major histocompatibility complex (MHC) presentation, as well as activate a desired immunological phenotype. Recently, a dual intracranial route of administration of CAR T cells has been applied to target IL-13Rα2 (interleukin-13 receptor subunit alpha-2), an overexpressing receptor in GBM, demonstrating salient initial response while reaffirming the challenges of GBM TME (Brown et al., 2016; Lim et al., 2018). Localized thermotherapies such as laser interstitial thermal therapy (LITT) have been explored in the treatment of GBM (van Solinge et al., 2022). LITT utilizes heat to destroy tumor tissue under the magnetic resonance imaging (MRI) guidance; it can either be combined with radiotherapy or serve as a viable alternative when conventional surgeries are deemed suboptimal (Thomas et al., 2016; Kamath et al., 2019; de Groot et al., 2022). Drugs could be administered in conjunction with other treatments. PLX3397 is a colony-stimulating factor-1 receptor (CSF1R) inhibitor that reduces microglia, tumor burden, and invasion in preclinical models (Butowski et al., 2016; Wolf et al., 2019). Cilengitide is an integrin inhibitor that reduces angiogenesis and shows promise in phase I and II studies (Nabors et al., 2007; Gilbert et al., 2012; Scaringi et al., 2012; Wolf et al., 2019). AQ4N is a potent bioreductive prodrug, which is selectively expressed in hypoxia-activated tumors (Patterson and McKeown, 2000; Albertella et al., 2008).

In addition to the aforementioned research directions, biomaterials, and engineered devices have been introduced to craft a variety of models that mimic the TME for better preclinical studies (Nakod et al., 2018; Wolf et al., 2019; Paolillo et al., 2021). Two-dimensional (2D) matrix models fabricate substrates with extracellular matrix (ECM) ligands and mechanical properties similar to the brain matrix (Xiao et al., 2017). 3D matrix models further expand on the dimensionality, incorporate materials (e.g., collagen, HA, Matrigel, and synthetic polymers), and may better capture the brain architecture compared to 2D models (Ananthanarayanan et al., 2011; Fernandez-Fuente et al., 2014; Xiao et al., 2017; Diao et al., 2019; Wolf et al., 2019; Paolillo et al., 2021). Combining the 2D patterning with 3D-like constraints gives rise to semi-3D (2.5D) models (Wolf et al., 2019). These models more faithful represent the tissue architecture and allow for better cell morphology studies (Wolf et al., 2019; Paolillo et al., 2021).

Using 2D, and 3D biomimetic models, and *in vivo* mouse models, Rubenich et al. investigated the role of isolated human neutrophils on the U87MG glioblastoma tumors. In the study, neutrophils were isolated and processed from healthy volunteers; immunohistochemistry staining results suggested that neutrophils mostly proliferate in the tumor periphery. A 2D glioma-neutrophil co-culture effectively proved that neutrophils can promote glioma development. It was also found that the contact between glioma and neutrophils positively reinforced glioma proliferation after 72 h and, notably, after 120 h. To uncover the mechanism of glioma-neutrophil crosstalk, researchers generated three-dimensional spheroids of the glioma and infiltrated them with a pool of neutrophils, a combination eliciting significantly faster glioma proliferation. The glioma morphology in this 3D culture was further verified by hematoxylin and eosin (HE) and Ki67 staining, with results supporting the idea that neutrophils can foster tumor progression within a regulated 3D environment. Overall, these investigations examined the close contacts between neutrophils and glioma under different models and led to the conclusion that neutrophils influence and promote tumor growth in TME.

Macrophages are another type of immune cell that plays a crucial role in GBM. Xing et al. retrieved data from the GEO database of patients with glioma and, upon filtering out doublet cells and analyzing the remaining cell clusters using R Software, identified macrophages as their interest. Macrophages primarily aggregate in the tumor core and exhibit a significantly increased oxidative stress activity. Further analysis identified *RXRA, RARA, MXI1, FOSL2*, and *BHLHE40* as the five most expressed transcription factors in macrophage oxidate stress activity. The study also highlighted the prominent role of the SPP1-CD44 receptor-ligand pair in macrophage communication with other cell types, particularly microglia, implying the latter's antagonistic role. A weighted co-expression network analysis (WGCNA) was subsequently applied, grouping genes that were expressed together in macrophages; two modules, M1 and M3, were identified as closely associated with macrophages. From these modules, a high-risk gene *MANBA*, and a low-risk gene *TCF12* were particularly relevant to patient survival. In a high *MANBA* level environment, researchers observed pro-tumor characteristics, such as promoted cell chemotaxis, humoral immune response, and increased response to chemokine. In contrast, *MANBA* knockdown resulted in saliently decreased invasiveness of GBM, further proving the supportive role of *MANBA* in GBM proliferation.

In addition to immune cells, genetic factors might as well promote glioma. Shen et al. employed pan-cancer analysis on schizophrenia-associated genes (*HTR2A, COMT*, and *PRODH*). Through a comprehensive statistical analysis, they revealed that these genes each demonstrated differential expression and significant effects across a variety of tumor types, but all three genes showed considerable correlation to the carcinogenesis and survival in glioblastoma and low-grade glioma. Since CD8^+^ T cells are known as crucial anti-tumor lymphocytes, researchers' results therefore suggested the importance of CD8^+^ T cells in patients' prognosis and management.

Greenlund et al. conducted a separate statistical analysis on the effects of conventional therapy (180–200 cGy per fraction) vs. hypofractionated radiotherapy (>200 cGy per fraction and 15 or fewer fractions). A retrospective cohort study, this research focused on the peripheral leukocyte of newly diagnosed GBM, taking for measurement the patients' complete blood counts (CBC) before, during, and after their chemoradiation treatments. Using these data, researchers were able to establish a prediction model that accounted for the temporal effects of treatments, as well as different baseline values of immune cells and patient blood counts. The result showed an increased monocyte concentration and a decreased lymphocyte concentration in patients treated with conventional therapy, as compared to hypofractionated therapy. This study not only implied the alterations in immunology profiles due to different radiotherapy schemes but also provided future radiotherapy directions.

Several other studies explored potential prognostic markers and therapeutic targets in glioma. Since telomeres are known to play an important role in lower-grade glioma (LGG) progression, the research group (Han et al.) detailed a telomere-tumor microenvironment (TM-TME) classifier method to enhance prognostic predictions in LGG. Researchers sampled data of patients with LGG from the cancer genome atlas (TCGA) and the Chinese glioma genome atlas (CGGA)databases, from which they applied the LASSO Cox regression model to derive telomere-associate genes and estimated immune cell compositions. These data allowed researchers to obtain TM and TME scores and construct a TM-TME classifier. To further account for single-cell nuances and gene interactions, researchers added RNA-seq and WGCNA to this classifier. Additionally, researchers used the Tumor Immune Dysfunction and Exclusion (TIDE) platform to predict immunotherapeutic outcomes in different tumor subgroups. Using Gene Ontology (GO) analysis, researchers determined that patients categorized as TM_low + TME_high had the most favorable prognosis and a better potential for immunotherapy responses, underscoring a potent personalized treatment avenue, while the TM_high + TME_low subgroup had poorer prognosis and worse immune response.

Xu et al. utilized the TCGA database of patients with glioma and identified 14 ferroptosis-related risk genes in glioblastoma multiforme. Their results showed that eight out of the 14 genes, especially *HBA1, GDF15*, and *NNMT*, were significantly overexpressed in the high-risk group, contributing to worsened patient prognosis and therefore noted as risk genes. In addition, single-cell analysis revealed that the ferroptosis-related genes, *AURKA, HSPB1*, and *NNMT*, were highly expressed in M2 macrophages. M2 macrophages are known to contribute to tumor progression. When risk genes were overexpressed, researchers also detected a high M2/M1 ratio and the transition from M1 to M2 in the TME, highlighting the complex dynamic of ferroptosis-macrophage polarization in GBM.

Lastly, studies by Zhou et al. and Guo et al. explored the potential effects and uses of disulfidptosis, a novel form of programmed cell death, in LGG. Zhou et al. obtained their LGG data from TCGA and CGGA, subjected to WGCNA and further statistical refinements, and eventually produced nine disulfidptosis-associated genes (DAG). These DAGs stratified patients with LGG into high-risk and low-risk groups, with the former group exhibiting poorer prognosis, distinct clinicopathological features, elevated regulatory T cells expression in TME, low frequencies of isocitrate dehydrogenase 1 (IDH1) mutations, and higher tumor mutation burden. After single-cell RNA sequencing (scRNA-seq) of the nine DAGs within the TME of various cell types, researchers also confirmed that *ABI3* predominantly expresses in malignant glioma; their follow-up knockdown experiment reinforced the role of *ABI3* in cell migration and invasion. On the other hand, Guo et al. sought to explore a new venue that incorporates disulfidptosis-related lncRNAs (DRlncRNAs) into glioma therapy. They utilized TCGA and GTEx data to identify 10 disulfidptosis-related genes (DRGs) across 34 cancer types. Two of these DRGs, *GYS1* and *RPN1*, showed higher expression in GBM than LGG, as well as demonstrated a relationship with high CD163 (M2 macrophage marker) expression in the subsequent immunohistochemistry analysis. The researchers also established a risk signature using eight DRlncRNAs by dividing patients into a high-risk and a low-risk group, where the high-risk group was associated with poor survival, more immune cell infiltration, and high tumor mutation burden (TMB). Patient survival was accurately predicted using a nomogram that combined the risk score with patients' clinical features. Functional analysis revealed the involvement of differentially expressed lncRNAs genes in processes such as extracellular matrix organization, focal adhesion, and pathways in cancers. Finally, the experiment showed that one of the lncRNAs in the risk signature, *LINC02525*, can be knocked down and lead to reduced glioma invasiveness and increased F-actin disulfidptosis.

The studies discussed in this editorial underline the factors leading to GBM progression and highlight potential treatments for this aggressive tumor. Therapies and drugs have shown promise in the early stages of studies, but their efficacies are frequently limited by the complexity of the human brain, among other factors. Biomimetic models have provided valuable insights into the TME, identifying neutrophils and macrophages as crucial players in GBM growth. Statistical data have shed light on the linkage of CD8^+^ T cells to carcinogenesis and patient survival, while a retrospective analysis explained how conventional radiotherapy results in higher monocyte and decreased lymphocyte concentrations. Among the recent updates, various prognostic markers have been identified, including TM-TME classifiers, ferroptosis-related genes, and disulfidptosis-associated genes and lncRNAs. These studies help us better understand the immense potential of a multidisciplinary approach, a necessary step to overcoming the challenges in GBM and improving patient outcomes.

## Author contributions

SX: Writing—original draft, Writing—review & editing. BQ: Writing—review & editing. CL: Writing—review & editing. YP: Writing—review & editing.

## References

[B1] Akiyama Y. Jung S. Salhia B. Lee S. Hubbard S. Taylor M.. (2001). Hyaluronate receptors mediating glioma cell migration and proliferation. J. Neurooncol. 53, 115–127. 10.1023/A:1012297132047 11716065

[B2] Albertella M. R. Loadman P. M. Jones P. H. Phillips R. M. Rampling R. Burnet N.. (2008). Hypoxia-selective targeting by the bioreductive prodrug AQ4N in patients with solid tumors: results of a phase I study. Clin. Cancer Res. 14, 1096–1104. 10.1158/1078-0432.CCR-07-4020 18281542

[B3] Ananthanarayanan B. Kim Y. Kumar S. (2011). Elucidating the mechanobiology of malignant brain tumors using a brain matrix-mimetic hyaluronic acid hydrogel platform. Biomaterials 32, 7913–7923. 10.1016/j.biomaterials.2011.07.005 21820737 PMC3159794

[B4] Bellail A. C. Hunter S. B. Brat D. J. Tan C. Van Meir E. G. (2004). Microregional extracellular matrix heterogeneity in brain modulates glioma cell invasion. Int. J. Biochem. Cell Biol. 36, 1046–1069. 10.1016/j.biocel.2004.01.013 15094120

[B5] Brown C. E. Alizadeh D. Starr R. Weng L. Wagner J. R. Naranjo A.. (2016). Regression of glioblastoma after chimeric antigen receptor T-cell therapy. N. Engl. J. Med. 375, 2561–2569. 10.1056/NEJMoa1610497 28029927 PMC5390684

[B6] Butowski N. Colman H. De Groot J. F. Omuro A. M. Nayak L. Wen P. Y.. (2016). Orally administered colony stimulating factor 1 receptor inhibitor PLX3397 in recurrent glioblastoma: an Ivy Foundation Early Phase Clinical Trials Consortium phase II study. Neuro Oncol. 18, 557–564. 10.1093/neuonc/nov245 26449250 PMC4799682

[B7] de Groot J. F. Kim A. H. Prabhu S. Rao G. Laxton A. W. Fecci P. E.. (2022). Efficacy of laser interstitial thermal therapy (LITT) for newly diagnosed and recurrent IDH wild-type glioblastoma. Neurooncol. Adv. 4:vdac040. 10.1093/noajnl/vdac040 35611270 PMC9122789

[B8] Diao W. Tong X. Yang C. Zhang F. Bao C. Chen H.. (2019). Behaviors of glioblastoma cells in *in vitro* microenvironments. Sci. Rep. 9:85. 10.1038/s41598-018-36347-7 30643153 PMC6331579

[B9] Fernandez-Fuente G. Mollinedo P. Grande L. Vazquez-Barquero A. Fernandez-Luna J. L. (2014). Culture dimensionality influences the resistance of glioblastoma stem-like cells to multikinase inhibitors. Mol. Cancer Ther. 13, 1664–1672. 10.1158/1535-7163.MCT-13-0854 24723451

[B10] Franceschi E. Tosoni A. Bartolini S. Mazzocchi V. Fioravanti A. Brandes A. A.. (2009). Treatment options for recurrent glioblastoma: pitfalls and future trends. Expert Rev. Anticancer Ther. 9, 613–619. 10.1586/era.09.23 19445578

[B11] Gilbert M. R. Kuhn J. Lamborn K. R. Lieberman F. Wen P. Y. Mehta M.. (2012). Cilengitide in patients with recurrent glioblastoma: the results of NABTC 03-02, a phase II trial with measures of treatment delivery. J. Neurooncol. 106, 147–153. 10.1007/s11060-011-0650-1 21739168 PMC4351869

[B12] Immonen A. Vapalahti M. Tyynela K. Hurskainen H. Sandmair A. Vanninen R.. (2004). AdvHSV-tk gene therapy with intravenous ganciclovir improves survival in human malignant glioma: a randomised, controlled study. Mol. Ther. 10, 967–972. 10.1016/j.ymthe.2004.08.002 15509514

[B13] Kamath A. A. Friedman D. D. Akbari S. H. A. Kim A. H. Tao Y. Luo J.. (2019). Glioblastoma treated with magnetic resonance imaging-guided laser interstitial thermal therapy: safety, efficacy, and outcomes. Neurosurgery 84, 836–843. 10.1093/neuros/nyy375 30137606 PMC6425465

[B14] Koshy M. Villano J. L. Dolecek T. A. Howard A. Mahmood U. Chmura S. J.. (2012). Improved survival time trends for glioblastoma using the SEER 17 population-based registries. J. Neurooncol. 107, 207–212. 10.1007/s11060-011-0738-7 21984115 PMC4077033

[B15] Lim M. Xia Y. Bettegowda C. Weller M. (2018). Current state of immunotherapy for glioblastoma. Nat. Rev. Clin. Oncol. 15, 422–442. 10.1038/s41571-018-0003-5 29643471

[B16] Nabors L. B. Mikkelsen T. Rosenfeld S. S. Hochberg F. Akella N. S. Fisher J. D.. (2007). Phase I and correlative biology study of cilengitide in patients with recurrent malignant glioma. J. Clin. Oncol. 25, 1651–1657. 10.1200/JCO.2006.06.6514 17470857 PMC3811028

[B17] Nakod P. S. Kim Y. Rao S. S. (2018). Biomimetic models to examine microenvironmental regulation of glioblastoma stem cells. Cancer Lett. 429, 41–53. 10.1016/j.canlet.2018.05.007 29746930

[B18] Ostrom Q. T. Gittleman H. Farah P. Ondracek A. Chen Y. Wolinsky Y.. (2013). statistical report: primary brain and central nervous system tumors diagnosed in the United States in 2006-2010. Neuro. Oncol. 15(Suppl 2), ii1–56. 10.1093/neuonc/not151 24137015 PMC3798196

[B19] Paolillo M. Comincini S. Schinelli S. (2021). *In vitro* glioblastoma models: a journey into the third dimension. Cancers 13:2449. 10.3390/cancers13102449 34070023 PMC8157833

[B20] Patterson L. H. McKeown S. R. (2000). AQ4N: a new approach to hypoxia-activated cancer chemotherapy. Br. J. Cancer 83, 1589–1593. 10.1054/bjoc.2000.1564 11104551 PMC2363465

[B21] Quail D. F. Joyce J. A. (2017). The microenvironmental landscape of brain tumors. Cancer Cell 31, 326–341. 10.1016/j.ccell.2017.02.009 28292436 PMC5424263

[B22] Rath B. H. Wahba A. Camphausen K. Tofilon P. J. (2015). Coculture with astrocytes reduces the radiosensitivity of glioblastoma stem-like cells and identifies additional targets for radiosensitization. Cancer Med. 4, 1705–1716. 10.1002/cam4.510 26518290 PMC4673998

[B23] Reardon D. A. Brandes A. A. Omuro A. Mulholland P. Lim M. Wick A.. (2020). Effect of nivolumab vs bevacizumab in patients with recurrent glioblastoma: the CheckMate 143 phase 3 randomized clinical trial. JAMA Oncol. 6, 1003–1010. 10.1001/jamaoncol.2020.1024 32437507 PMC7243167

[B24] Scaringi C. Minniti G. Caporello P. Enrici R. M. (2012). Integrin inhibitor cilengitide for the treatment of glioblastoma: a brief overview of current clinical results. Anticancer Res. 32, 4213–4223. 23060541

[B25] Schuster J. Lai R. K. Recht L. D. Reardon D. A. Paleologos N. A. Groves M. D.. (2015). A phase II, multicenter trial of rindopepimut (CDX-110) in newly diagnosed glioblastoma: the ACT III study. Neuro Oncol. 17, 854–861. 10.1093/neuonc/nou348 25586468 PMC4483122

[B26] Stupp R. Mason W. P. van den Bent M. J. Weller M. Fisher B. Taphoorn M. J.. (2005). Radiotherapy plus concomitant and adjuvant temozolomide for glioblastoma. N. Engl. J. Med. 352, 987–996. 10.1056/NEJMoa043330 15758009

[B27] Thomas J. G. Rao G. Kew Y. Prabhu S. S. (2016). Laser interstitial thermal therapy for newly diagnosed and recurrent glioblastoma. Neurosurg. Focus 41:E12. 10.3171/2016.7.FOCUS16234 27690657

[B28] van Solinge T. S. Nieland L. Chiocca E. A. Broekman M. L. D. (2022). Advances in local therapy for glioblastoma - taking the fight to the tumour. Nat. Rev. Neurol. 18, 221–236. 10.1038/s41582-022-00621-0 35277681 PMC10359969

[B29] Weller M. Butowski N. Tran D. D. Recht L. D. Lim M. Hirte H.. (2017b). Rindopepimut with temozolomide for patients with newly diagnosed, EGFRvIII-expressing glioblastoma (ACT IV): a randomised, double-blind, international phase 3 trial. Lancet Oncol. 18, 1373–1385. 10.1016/S1470-2045(17)30517-X 28844499

[B30] Weller M. Kaulich K. Hentschel B. Felsberg J. Gramatzki D. Pietsch T.. (2014). Assessment and prognostic significance of the epidermal growth factor receptor vIII mutation in glioblastoma patients treated with concurrent and adjuvant temozolomide radiochemotherapy. Int. J. Cancer 134, 2437–2447. 10.1002/ijc.28576 24614983

[B31] Weller M. van den Bent M. Tonn J. C. Stupp R. Preusser M. Cohen-Jonathan-Moyal E.. (2017a). European Association for Neuro-Oncology (EANO) guideline on the diagnosis and treatment of adult astrocytic and oligodendroglial gliomas. Lancet Oncol. 18, e315–e329. 10.1016/S1470-2045(17)30194-8 28483413

[B32] Wheeler L. A. Manzanera A. G. Bell S. D. Cavaliere R. McGregor J. M. Grecula J. C.. (2016). Phase II multicenter study of gene-mediated cytotoxic immunotherapy as adjuvant to surgical resection for newly diagnosed malignant glioma. Neuro Oncol. 18, 1137–1145. 10.1093/neuonc/now002 26843484 PMC4933478

[B33] Wolf K. J. Chen J. Coombes J. Aghi M. K. Kumar S. (2019). Dissecting and rebuilding the glioblastoma microenvironment with engineered materials. Nat. Rev. Mater 4, 651–668. 10.1038/s41578-019-0135-y 32647587 PMC7347297

[B34] Xiao W. Sohrabi A. Seidlits S. K. (2017). Integrating the glioblastoma microenvironment into engineered experimental models. Future Sci. OA 3:FSO189. 10.4155/fsoa-2016-0094 28883992 PMC5583655

[B35] Yoo K. C. Suh Y. An Y. Lee H. J. Jeong Y. J. Uddin N.. (2018). Proinvasive extracellular matrix remodeling in tumor microenvironment in response to radiation. Oncogene 37, 3317–3328. 10.1038/s41388-018-0199-y 29559744

